# Counting stillbirths and achieving accountability: A global health priority

**DOI:** 10.1371/journal.pmed.1002364

**Published:** 2017-08-01

**Authors:** Zulfiqar A. Bhutta

**Affiliations:** Robert Harding Chair in Global Child Health and Policy, Centre for Global Child Health, The Hospital for Sick Children, Toronto, Canada

## Abstract

In a Perspective, Zulfiqar Bhutta discusses the importance of regular and timely reporting on stillbirths.

Although the millennium development goals (MDGs) focused on maternal and child health and survival at inception, the importance of newborn survival to achievement of MDG 4 was soon well recognized [1]. Over the last several years, the relative importance of stillbirths and their links to interventions to address maternal and newborn health have also been underscored [2]. Despite this advocacy, the estimated 2.6 million stillbirths globally largely remain a hidden issue on the global policy platform, with little to no awareness for action at the country level [2]. It took much effort towards the end of the MDG period to have stillbirth rates included as one of the 16 key indicators for monitoring progress for the global strategy for women, children, and adolescents [3].

We need better data than modeled estimates to better define the burden and etiology of stillbirths from representative population-based studies or vital registration systems. The latter are relatively uncommon as the source of information in low- and middle-income settings. In this week's *PLOS Medicine*, Dandona and colleagues [4] underscore the importance of stillbirths in a population-based survey of Bihar (India). The study was based on verbal autopsies conducted on 1,132 stillbirths identified among 100,000 households over a 38-month period. Their identified incidence rate of stillbirths of 21.2 per 1,000 births (95% CI 19.7–22.6) is very close to the modeled estimated rate of 22 per 1,000 births [5]. In a little over a third of these stillbirths, no cause could be identified, whereas obstetric complications and hemorrhage were associated with 30% of stillbirths. These findings are broadly consonant with the limited information on etiology from other studies of stillbirths [5,6]. In a large verbal autopsy–based analysis of 1,285 stillbirths across a national sample of 95,000 households in the Pakistan demographic and health survey in 2006, 33.5% of antepartum stillbirths and 20.9% of intrapartum stillbirths did not have a clear cause of death [7].

Many of the findings reported by Dandona et al [4] are well recognized risk factors for poor pregnancy outcomes, such as the association of stillbirths with poor quality of antenatal care (no antenatal care in 41% and 52% of urban or rural samples, respectively), small size of the fetus (25% and 38%), maternal fever in about 15%–16%, and evidence of infection in 15%–20% of subjects. No information is provided on the proportion of births among younger adolescents (<18 years), an important factor in the context of India, where adverse birth outcomes are associated with young maternal age [8]. Similarly, we do not have information specific on access to caesarean section deliveries in the cohort, as limited access to emergency caesarean sections is a recognized risk factor for stillbirths at the population level [5]. Given the lack of corresponding data on general births in Bihar or neonatal deaths, it is difficult to extrapolate the findings to beyond the stillbirth cohort. However, the risk factors are well known and do provide information relevant for policy. The authors relied on verbal narratives in their analysis of risk factors and given the link with health system performance and responsiveness, the findings also underscore the failings within the health system to provide quality maternal care, especially around childbirth. Future research studies might also include concurrently administered social autopsy instruments to better understand pathways to care during pregnancy and childbirth, potential delays, and the antecedents of intrapartum stillbirths. These social autopsy modules and instruments are being increasingly deployed at the population level in addition to standard narratives with verbal autopsy instruments. Over the last few years, the WHO verbal autopsy instruments and methodology for implementation have also been improved and the stillbirth module therein validated prospectively [9]. The WHO instrument on verbal autopsies has now been updated for 2016 and is undergoing further field testing with automated analysis methods.

The reported male predominance among the stillbirths is interesting, and the differential is higher than the slight male predominance previously reported in the literature [10]. Given the known association of gender imbalances associated with selective female feticide [11], one wonders if the male-to-female ratio among stillbirths could have been affected by such factors within the population cohort. This is a subject for future studies using routine information systems and concurrent information at the population level on reported miscarriages and abortions.

Notwithstanding the need for more granular information on the timing and risk factors for stillbirths, these findings from Bihar underscore the importance of focusing on stillbirths in addition to newborn deaths as India embarks on its strategy for improving newborn and birth outcomes. The India Newborn Action Plan includes strategies to reduce preventable newborn deaths and stillbirths to less than 10 per 1,000 births by the year 2030 [12]. This is based on the implementation of evidence-based interventions across the continuum of care and tracking of key indicators, including stillbirth and intrapartum stillbirth rates [12]. The focus is on implementation. Although the interventions needed to impact maternal, fetal, and newborn health and the delivery strategies to implement them are well described [13], much more work is needed in scaling up such interventions through appropriate delivery platforms focused on balancing supply and demand and reaching the marginalized populations [14]. This could consist of strategies to include stillbirths in the repertoire of activities by community health and outreach workers engaged in maternal and newborn health as well as outcome evaluations.

Regular and timely reporting on stillbirths, especially intrapartum stillbirths, is a priority. Not only is this needed to reduce current disparities in care, it is also imperative that intrapartum stillbirths are recognized as an extension of early neonatal deaths, especially those associated with intrapartum complications and birth asphyxia. Much more needs to be done to include stillbirths in national reporting systems and to improve the quality of vital registration programs as well as classification systems used to categorize stillbirths [15]. The inclusion of stillbirth rate estimation among the key indicators for the global strategy for women, children, and adolescents is a step in the right direction but needs to be complemented by robust implementation and annual reporting.
